# Location Is Everything: Influence of His-Tag Fusion Site on Properties of Adenylosuccinate Synthetase from *Helicobacter pylori*

**DOI:** 10.3390/ijms25147613

**Published:** 2024-07-11

**Authors:** Marija Zora Mišković, Marta Wojtyś, Maria Winiewska-Szajewska, Beata Wielgus-Kutrowska, Marija Matković, Darija Domazet Jurašin, Zoran Štefanić, Agnieszka Bzowska, Ivana Leščić Ašler

**Affiliations:** 1 Department of Chemistry, Faculty of Science, University of Zagreb, Horvatovac 102a, HR-10000 Zagreb, Croatia; mmiskovic@stud.biol.pmf.hr; 2 Division of Biophysics, Institute of Experimental Physics, Faculty of Physics, University of Warsaw, Pasteura 5, 02-093 Warsaw, Poland; mi.wojtys@fuw.edu.pl (M.W.); maria.winiewska@fuw.edu.pl (M.W.-S.); beata.wielgus-kutrowska@fuw.edu.pl (B.W.-K.); 3 Institute of Biochemistry and Biophysics, Polish Academy of Sciences, Pawinskiego 5a, 02-106 Warsaw, Poland; 4 Division of Organic Chemistry and Biochemistry, Ruđer Bošković Institute, Bijenička cesta 54, HR-10000 Zagreb, Croatia; marija.matkovic@irb.hr; 5 Division of Physical Chemistry, Ruđer Bošković Institute, Bijenička cesta 54, HR-10000 Zagreb, Croatia; darija.domazet.jurasin@irb.hr (D.D.J.); zoran.stefanic@irb.hr (Z.Š.)

**Keywords:** adenylosuccinate synthetase, *Helicobacter pylori*, His-tag, enzyme kinetics, protein structure, protein stability

## Abstract

The requirement for fast and dependable protein purification methods is constant, either for functional studies of natural proteins or for the production of biotechnological protein products. The original procedure has to be formulated for each individual protein, and this demanding task was significantly simplified by the introduction of affinity tags. *Helicobacter pylori* adenylosuccinate synthetase (AdSS) is present in solution in a dynamic equilibrium of monomers and biologically active homodimers. The addition of the His_6_-tag on the C-terminus (C-His-AdSS) was proven to have a negligible effect on the characteristics of this enzyme. This paper shows that the same enzyme with the His_6_-tag fused on its N-terminus (N-His-AdSS) has a high tendency to precipitate. Circular dichroism and X-ray diffraction studies do not detect any structural change that could explain this propensity. However, the dynamic light scattering, differential scanning fluorimetry, and analytical ultracentrifugation measurements indicate that the monomer of this construct is prone to aggregation, which shifts the equilibrium towards the insoluble precipitant. In agreement, enzyme kinetics measurements showed reduced enzyme activity, but preserved affinity for the substrates, in comparison with the wild-type and C-His-AdSS. The presented results reinforce the notion that testing the influence of the tag on protein properties should not be overlooked.

## 1. Introduction

Proteins are the most abundant macromolecules in living organisms. They participate in various biological events, such as maintaining the structures and properties of organisms, catalyzing chemical reactions, transporting nutrients and metabolic wastes, participating in the body’s immune defense, intracellular redox reaction, electron transfer, learning, and memory [1]. At the dawn of recombinant DNA technology, the focus of research was on DNA and genes coding for components of cells. However, in the post-genomic era, the focus shifted from high-throughput analysis of genome sequences to functional and structural studies of the proteins they encode. In addition to that, the use of proteins in biotechnology and as biotherapeutic medicines is constantly on the rise [2]. Therefore, the demand for fast and reliable procedures for obtaining large quantities of highly purified proteins is significant.

There are numerous techniques available for protein purification, each with its own advantages and disadvantages [1]. The purification procedure normally has to be custom-tailored for each protein, as it depends on protein properties, as well as the purpose of protein production [3]. Optimization of the procedure can take a long time and requires a significant amount of the sample; hence, the protein purification was (and often still is) considered as the bottleneck of the protein research. Affinity tags changed the field of protein purification at the end of the 1980s with the work of Hochuli et al. [4], who introduced the engineered histidine affinity handles and a special matrix (Ni(II)-nitrilotriacetic acid, Ni-NTA) [5]. Since then, affinity tags have proven to be tremendously effective tools for a wide variety of applications, primarily in shortened protein purification protocols, but also in the immobilization of proteins for display on a surface, in the detection and quantification of target proteins, in the analysis of protein–ligand and protein–protein interactions, and more recently in bioreactors for multistep enzymatic reactions and bioadsorbents for the extraction or degradation of toxic contaminants [6]. Affinity tags range in size from short peptides less than 1 kDa to large proteins (40 kDa and more). They are often found to be beneficial for the properties of recombinant construct—to increase the expression level and solubility, to simplify the (re)folding and enhance its efficiency, or to prevent proteolysis [7].

Immobilized metal affinity chromatography (IMAC) is a separation technique that uses metal ions chelated on solid chromatographic supports. These metal ions serve as affinity ligands for various proteins, making use of coordinative binding of naturally occurring amino acid residues exposed on the surface or residues added to the protein by genetic engineering [8]. The most commonly used metal ions are Ni^2+^ (on a Ni-NTA resin) and Co^2+^ (on a Co-CMA resin, TALON), and binding of proteins is achieved through their fusion with the His-tag (most often comprising six consecutive histidine residues). Today, IMAC is routinely used for the purification of proteins for various purposes, and it is generally thought that the small size and neutral charge of the His-tag do not influence the structure and function of the protein [1,7]. However, following the initial advice regarding the proper His-tag attachment site [5], more and more reports are published stating the negative effect of His-tag on protein’s stability, oligomerization state, structure, dynamics, function, and activity [9]. The introduction of the His-tag on the protein’s C-terminus even changed the processing of its N-terminus in the case of β-lactamase from the bacterium *Bacillus licheniformis* [10], while the N-terminal His-tag was used to tailor lipase LipC12 activity from hydrolysis to esterification [11].

We have committed our efforts to the characterization of enzymes that participate in the synthesis of purines, primarily in the bacterium *Helicobacter pylori*. This dangerous pathogen is estimated to infect 50% of the world’s population. It is involved in the development of serious diseases, including peptic ulcer, gastric cancer, and gastric mucosa-associated lymphoid tissue lymphoma [12]. As the number of antibiotic-resistant strains of *H. pylori* is on the rise, there is an urgent need to discover new courses of eradication of this bacterium [13,14]. The enzymes involved in purine biosynthesis present possible targets for new antibacterial drugs [15], and this is especially emphasized in *H. pylori* which lacks several enzymes of de novo purine synthesis and relies solely on the purine salvage pathway [16]. Adenylosuccinate synthetase (AdSS) participates in both biosynthesis and salvage pathways of purines. It is shown to be necessary for the survival of *H. pylori* [16], therefore its inhibitors have the potential to be new drugs against this pathogen. AdSS catalyzes the formation of adenylosuccinate (succinyl adenosine monophosphate, AMPS) from inosine monophosphate (IMP) and aspartate (Asp), accompanied by hydrolysis of one molecule of guanosine triphosphate (GTP) in the presence of magnesium ions:

IMP + Asp + GTP(Mg^2+^) ‹–› AMPS + GDP + P_i_

We have previously purified and characterized wild-type (WT) *H. pylori* AdSS [17]. In order to simplify the purification of larger amounts of protein for crystallization, we added His_6_-tag to AdSS—either on the protein’s N-terminus (N-His-AdSS) followed by a thrombin recognition site or on the protein’s C-terminus (C-His-AdSS) without any protease recognition site. We demonstrated a negligible effect of the C-terminal tag on the enzyme’s behavior and solved crystal structures of the fully ligated protein (with guanosine diphosphate (GDP), 6-phosphoryl-inosine monophosphate (6-P-IMP), Asp analog hadacidin (Had), and Mg^2+^) and the protein–IMP complex [18]. In this paper, we describe the detailed analysis of the catalytic and structural properties of *H. pylori* N-His-AdSS, in comparison to the previously characterized variants.

## 2. Results

### 2.1. Overexpression and Purification of N-His-AdSS in Comparison to C-His-AdSS

**Figure 1:**
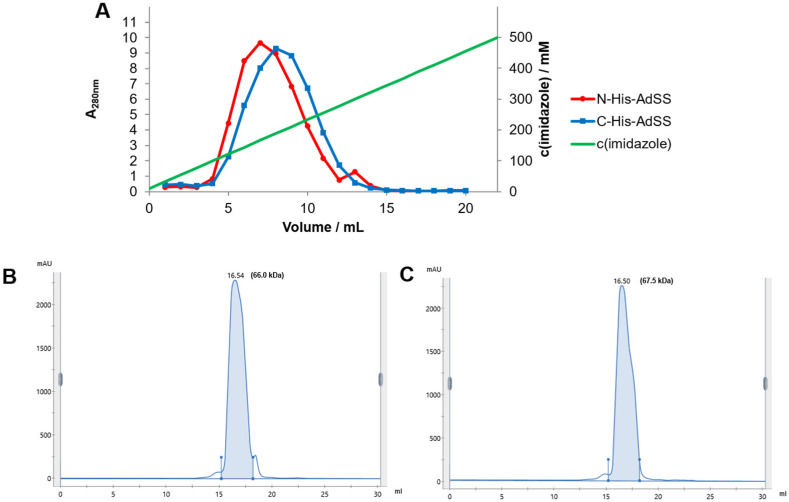
Purification of N-His-AdSS and C-His-AdSS from *H. pylori*. (**A**)—elution of both enzyme variants from affinity chromatography column, (**B**)—SEC chromatogram of N-His-AdSS, and (**C**)—SEC chromatogram of C-His-AdSS. Elution volumes (in mL) and molecular weights (in kDa) of AdSS enzyme variants are noted on SEC chromatograms.

**Figure 2:**
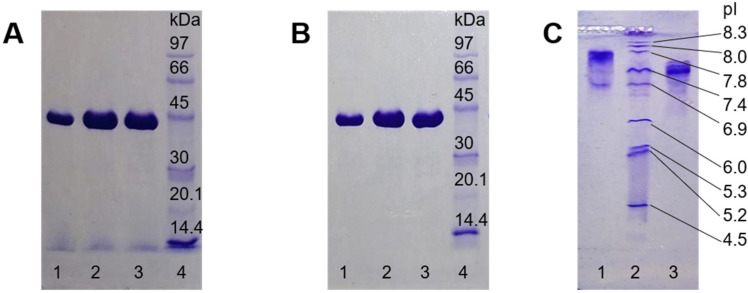
Purity of purified *H. pylori* AdSS enzyme variants. (**A**)—SDS-PAGE (12.5%) of N-His-AdSS SEC fractions (from the peak shaded blue in Figure 1B), (**B**)—SDS-PAGE (12.5%) of C-His-AdSS SEC fractions (from the peak shaded blue in Figure 1C), and (**C**)—IEF of purified N-His-AdSS (lane 1) and C-His-AdSS (lane 3). Lanes 4 in SDS-PAGE gels—LMW-SDS Marker Kit, lane 2 in IEF gel—IEF Marker 3–10, molecular masses and isoelectric points of markers are noted on the gels.

*Helicobacter pylori* adenylosuccinate synthetase (AdSS) variants with His_6_-tag attached to either N- or C-terminus were purified simultaneously utilizing the same procedure. Both enzymes were induced well with IPTG (isopropyl β-D-thiogalactopyranoside) and were expressed in good yield (~40 mg of protein per 1 L of bacterial culture). The proteins were purified as previously described [18]: by affinity chromatography (AC) and size exclusion chromatography (SEC). However, as N-His-AdSS has a higher pI than C-His-AdSS (as calculated by the ProtParam tool on Expasy.org, Table 1), the pH of the buffer for AC was lowered for this enzyme variant. Elution from the AC column was performed with the linear gradient of imidazole, and only a slight difference in the elution profile was observed. Namely, the protein peak eluted at 166 mM imidazole for N-His-AdSS and at 188 mM imidazole for C-His-AdSS (Figure 1). SEC chromatograms also looked very similar for both enzyme variants, with elution volumes of 16.54 mL for N-His-AdSS and 16.50 mL for C-His-AdSS (Figure 1). Final protein preparations appeared homogeneous on the SDS-PAGE (Figure 2).

Elution volumes from SEC for both enzyme variants correlate with the molecular weight (MW) of 66–68 kDa (Figure S1), i.e., between a monomer and a dimer of AdSS. This suggests the existence of a dynamic equilibrium between these two oligomeric forms of the enzyme, confirmed previously for the WT and C-His-AdSS [17,18]. Even though the shape of SEC peaks is not perfectly symmetrical, probably due to the high protein concentrations in the samples loaded (>60 mg/mL), the elution volume itself should not be significantly affected by this. A very similar elution volume (~16.30 mL, corresponding to ~76 kDa) was observed several times before with C-His-AdSS loaded in lower concentrations. Monomer molecular masses estimated from the SDS-PAGE gels agree very well with masses calculated from the protein sequences—47.8 and 46.1 kDa for N-His-AdSS and C-His-AdSS, respectively.

Purified AdSS variants were observed as not-so-sharp protein bands on isoelectric focusing (IEF), in the pI range 3–9 (Figure 2). However, for N-His-AdSS the most prominent band is approximately at pI = 7.8, and for C-His-AdSS the most prominent band is approximately at pI = 7.4. Those values correlate well with the isoelectric point (pI) values predicted from the protein sequence (7.94 and 7.22, respectively, see Table 1).

### 2.2. Stability of N-His-AdSS in Comparison to C-His-AdSS and WT AdSS

**Figure 3:**
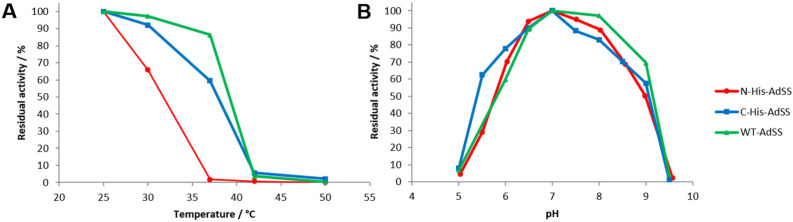
Temperature (**A**) and pH (**B**) stability profiles of N-His-AdSS, C-His-AdSS, and WT AdSS from *H. pylori*. The enzyme in 0.5 mg/mL concentration was incubated for one hour in 20 mM Hepes-NaOH buffer pH 7.7 at each temperature point (**A**) or for one hour in Britton–Robinson universal buffer of different pH at 25 °C (**B**).

The purification of both His-tagged AdSS variants was performed as fast as possible, however some precipitation of N-His-AdSS samples was observed. Therefore, the stability of this enzyme was checked under various pH and temperature conditions. Measurements show (Figure 3) that already after incubation for one hour at 30 °C N-His-AdSS loses ~35% of its activity (while C-His-AdSS and WT AdSS keep ≥90% of activity at this temperature [18]). Incubation of N-His-AdSS at 37 °C almost completely inactivates this variant of the enzyme (while C-His-AdSS and WT AdSS keep ~60% and ~85%, respectively, of activity at this temperature [18]). The pH stability range of N-His-AdSS is similar to those of C-His-AdSS and WT AdSS, namely 6.5–8.0 (Figure 3). Throughout our work, N-His-AdSS proved to be very unstable even when kept on ice, so for each measurement series a fresh aliquot was withdrawn from −80 °C in order to have reliable results.

As precipitation during work with N-His-AdSS was observed, dynamic light scattering (DLS) was employed to assess the homogeneity of the prepared samples. The DLS measurements confirm the uniformity of WT AdSS and C-His-AdSS samples, as only monomodal size distributions of protein particles are observed with an average hydrodynamic diameter (dh) of 6.0 ± 0.2 and 6.1 ± 0.3 nm, respectively (Figure S2). According to the Malvern Zetasizer software calculator for globular proteins, these dh values correspond to ~44 kDa. On the contrary, N-His-AdSS displays bimodal size distributions with two equally represented particle distributions having hydrodynamic diameters of 6.7 ± 0.2 nm (~57 kDa) and 253 ± 26 nm (Figure S2). Furthermore, the polydispersity index (PDI) for N-His-AdSS samples is significantly higher, equaling 0.9, compared to the measured samples of two other enzyme variants, which have a maximal PDI of 0.3.

To further compare all three enzyme variants, and also to check if the presence of NaCl affects their stability, nanoDSF (Low-Volume Differential Scanning Fluorimetry) measurements were performed (Table 2). As presented previously [18], in the presence of 150 mM NaCl the thermal stability of WT and C-His-AdSS differs only slightly. Similar behavior is observed now for the N-His-AdSS variant. An increase in NaCl concentration to 300 mM further stabilizes all three variants of the protein. However, without the addition of salt, both His-tagged variants are significantly less stable than the WT protein. Interestingly, in such conditions, stability is concentration-dependent, and all three variants are more stable at higher concentrations. The DSF results show that the presence of NaCl increases the middle-point temperature of the thermal unfolding (T_m_). However, confirming DLS results, in the DSF measurements a higher tendency to aggregation of the N-His-AdSS variant is observed, i.e., a lower onset aggregation temperature (T_turbdity_) than in the case of two other enzyme forms.

**Figure 4:**
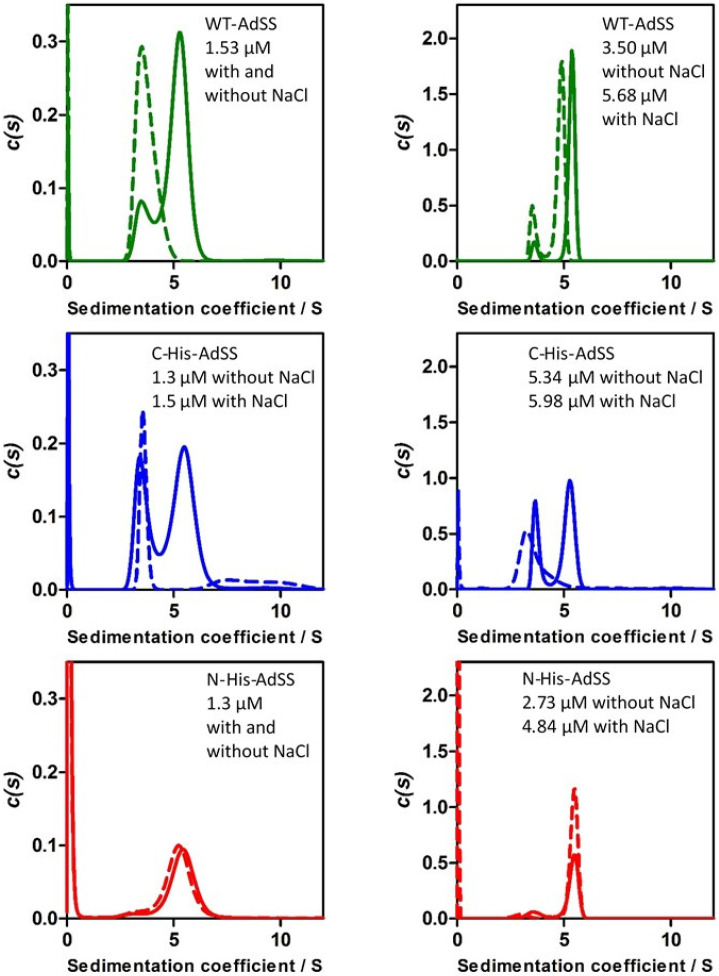
Sedimentation coefficient distribution, c(s), obtained for three *H. pylori* AdSS variants in the AUC sedimentation velocity experiments: wild type (**top**, green), C-His-AdSS (**middle**, blue), and N-His-AdSS (**bottom**, red). The AUC experiment was performed at 20 °C, in 20 mM Hepes-NaOH buffer with 1 mM TCEP, without (solid line, pH 6.79) or with (dashed line, pH 6.87) 150 mM NaCl. The experiment was run at 42,000 rpm, with the absorbance detection at 225 nm (**left** panels) and 230 nm (**right** panels).

It is known that AdSS exists in solution in a dynamic equilibrium of a monomer and a dimer, and its biologically active form is a dimer [17,18,19]. Therefore, in order to determine the distribution of all three AdSS variants between monomeric and oligomeric forms, analytical ultracentrifugation (AUC) experiments were performed. The influence of the enzyme concentration and the presence of NaCl was checked (Figure 4, Table 3). The theoretical value of the sedimentation coefficient of *H. pylori* AdSS monomer is s_monomer_ = 3.53 S, while for a dimer is s_dimer_ = 5.60 S (see Table 3 footnote for the methods of calculation used).

In agreement with the previous experiments [17,18], in the absence of NaCl, WT and C-His-AdSS are detected, regardless of the concentration studied, predominantly as a mixture of monomers and dimers in a dynamic equilibrium (Figure 4, upper and middle panels). By striking contrast, practically only dimer is detected in the case of N-His-AdSS. However, although the starting enzyme concentrations were the same, the height of the peak is much lower than in the case of two other enzyme variants (Figure 4), indicating that a substantial fraction of the protein aggregated and is not present in the solution. Since only the residual content of the monomeric form of N-His-AdSS is detected in the AUC experiment, the most probable explanation is that the monomer of this enzyme variant is much more prone to aggregation than its dimeric form, and also more than the monomeric forms of the WT AdSS and C-His-AdSS. The presence of NaCl has no influence on the oligomeric forms of the N-His-AdSS detected in the AUC experiment—again, practically only the peak corresponding to the dimer is visible, but it is reduced compared to the same peak in the WT AdSS and C-His-AdSS AUC experiments. To the contrary, in the case of WT and C-His-AdSS, the presence of NaCl shifts the equilibrium to the monomeric enzyme form, which, however, remains in solution. These monomers do not show a pronounced tendency for aggregation and precipitation, although some small fraction of C-His-AdSS in the presence of salt forms some larger but dissolved aggregates (Figure 4, middle left panel). The AUC data clearly show that the location of the His-tag has a great impact on the behavior of AdSS in solution, namely on the properties of the monomer, its tendency for aggregation and precipitation, and as a consequence, on the equilibrium of the oligomeric forms of the enzyme remaining in solution.

### 2.3. Kinetic Properties of N-His-AdSS in Comparison to C-His-AdSS

Specific activity was measured for both His-tagged enzyme variants in the standard assay mixture (0.06 mM GTP, 0.15 mM IMP, 5 mM Asp, 1 mM MgCl_2_, 20 mM Hepes-NaOH pH 7.7, followed for 3 min at 280 nm), immediately after purification. Specific activity for N-His-AdSS was 0.54 ± 0.19 U/mg, and for C-His-AdSS 1.04 ± 0.12 U/mg (average value of at least three repetitions). Even though specific activity (and, related to it, maximal reaction velocity) was significantly lower for N-His-AdSS than for C-His-AdSS, Michaelis–Menten constants for all three substrates were in the same range as those of C-His-AdSS and WT AdSS (Table 4).

### 2.4. Secondary Structure of N-His-AdSS in Comparison to C-His-AdSS

**Figure 5:**
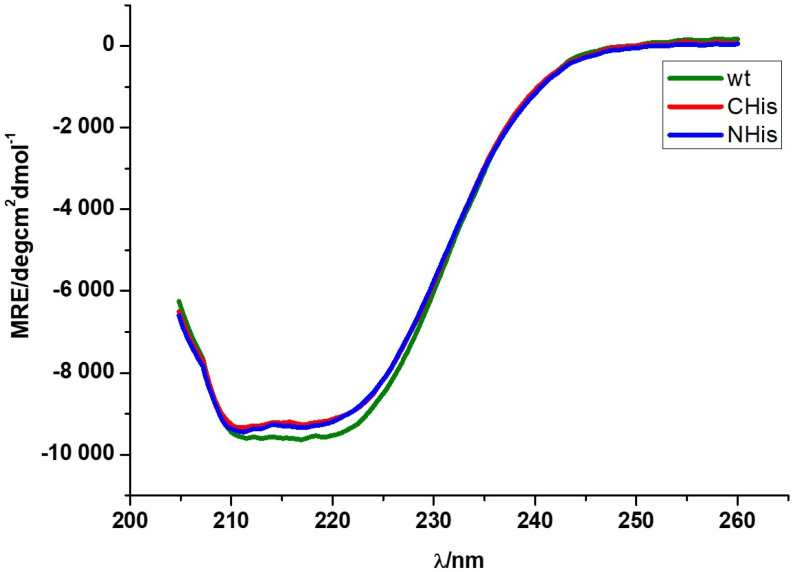
Averaged CD isothermal spectra taken for solutions of C-His-AdSS, N-His-AdSS, and WT AdSS from *H. pylori* (3 replicas) at concentration of 10 µM in SEC buffer. The curves were smoothed as described in Materials and Methods.

In order to inspect whether the propensity to aggregate and precipitate can be attributed to some structural change in N-His-AdSS, structural features of this enzyme variant were investigated. The secondary structure composition of the freshly purified N-His-AdSS and C-His-AdSS was determined by circular dichroism (UV-CD) and spectra compared to those of the WT AdSS. The CD spectra are shown in Figure 5 and depict a well-preserved secondary structure in C-His and N-His enzyme variants when compared to that of the WT AdSS. These visual inspections were confirmed by calculating the secondary structure elements percentage in each enzyme variant (Table 5) using the BeStSel online software [22] as described in Materials and Methods. No significant difference that could be correlated to the propensity of N-His-AdSS to aggregate was observed.

### 2.5. Crystal Structure of N-His-AdSS

**Figure 6:**
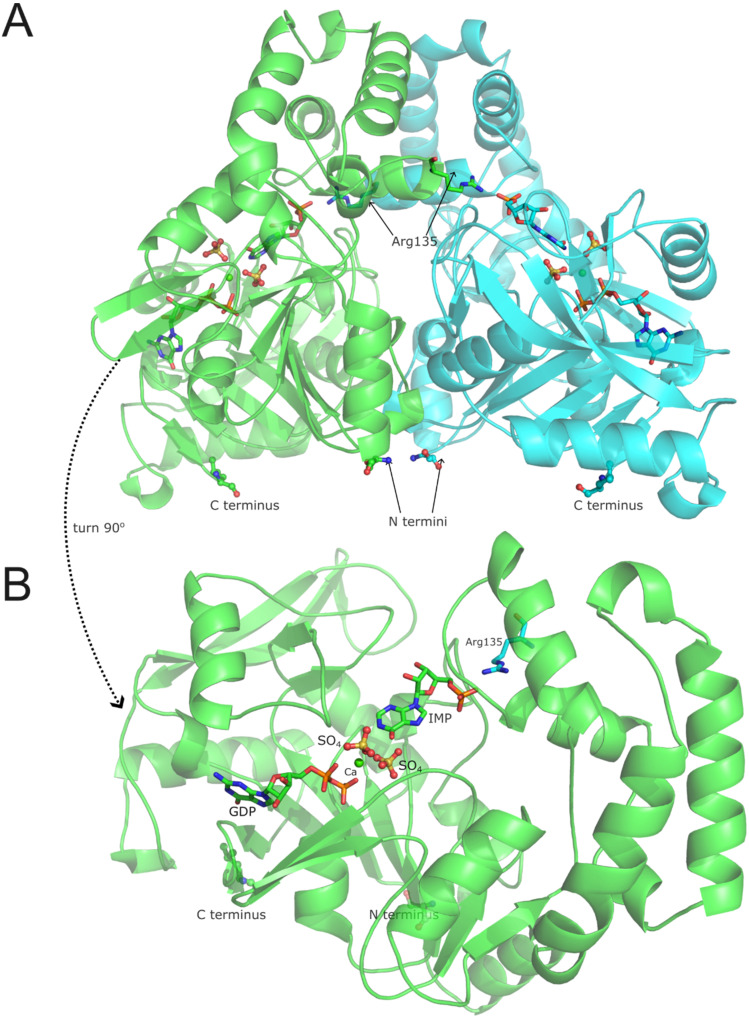
The overall structure of the N-His-AdSS dimer. (**A**) The positions of N and C termini are indicated on the bottom part of the protein, and it can be noted that all of them are on the surface of the protein, and N termini are quite close to one another. The position of Arg135 is also shown in both monomers and in each monomer, this amino acid makes hydrogen bonds to the IMP molecule located in the other monomer’s active site. (**B**) One subunit is shown with positions of the substrates and other ligands present in the active site.

Finally, we were able to determine the 3D structure of N-His-AdSS (coordinates deposited in ProteinDataBank, PDB, under code 9F17). The overall structure is nearly identical to the one determined for C-His-AdSS (PDB code 6ZXQ [18]), with RMSD between them equaling only 0.229 Å (as calculated with the align tool in PyMOL, version 3.0.0). Two monomers of the enzyme occupy the asymmetric unit, forming a catalytically active dimer, with the Arg135 side chain participating in building the active site of the neighboring monomer (Figure 6). Similar to the 6ZXQ structure of C-His-AdSS, the full length of the His-tag handle is not observed in the electron density maps. In the N-His-AdSS electron density map, only Ser and His upstream of Met1 are positioned in the structure but are not very well defined.

**Figure 7:**
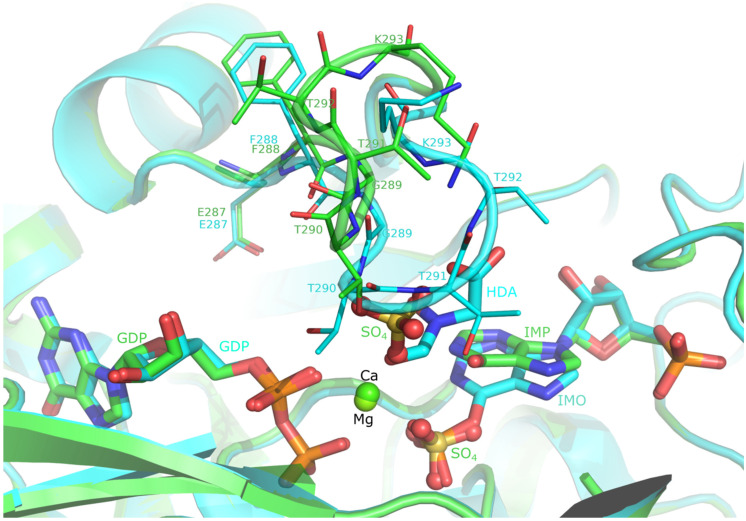
The active site of N-His-AdSS (structure and labels in green) compared to the active site of C-His-AdSS (structure and labels in cyan, PDB code 6ZXQ). It is visible that the GDP molecule occupies exactly the same position in both structures (left). However, the transformation from IMP to 6-P-IMP (IMO, right) causes a noticeable tilt of the plane of inosine. There are two SO_4_ molecules (shown in ball and stick) visible in the maps of the N-His-AdSS structure: one is situated exactly at the position of 6-P_i_ group of 6-P-IMP molecule (IMO, bottom), while the other is coordinated on the central metal ion from the side where the Had (HDA) molecule is coordinated (center). There is a very substantial drift away from the active site of the Asp-binding loop (residues 289–294, top).

N-His-AdSS enzyme was incubated with GTP, IMP, Had (hadacidin, analog of Asp), and MgCl_2_ during crystallization, just like the C-His-AdSS variant was [18]. However, clearly defined electron density for the Had molecule (HDA in the PDB denotation) is not visible in the structure (Figure 7). The shape of the electron density blob found in the approximate Asp-binding site of N-His-AdSS fits one sulfate ion, which could originate from the crystallization solution containing 0.2 M ammonium sulfate. One additional sulfate ion is found in the active site exactly at the position, which is occupied by the 6-P_i_ group of 6-P-IMP (IMO in the PDB denotation) in the structure of the C-His-AdSS variant. In accordance with the missing Had molecule, in the N-His-AdSS structure, the Asp-binding loop (residues 289–294) is moved away from the active site (Cα of Thr291 moved by 6.1 Å) and is poorly defined in the electron density. The electron density blob in the supposed Mg-binding site did not fit the Mg ion, so the Ca ion was placed there instead, as it made a much better match. Ca ions presumably originate from one of the used chemicals.

Ligands present in the active site of N-His-AdSS (Ca^2+^, GDP, and IMP) are anchored similarly to their counterparts in the 6ZXQ structure of the C-His-AdSS. The calcium ion is coordinated with six oxygen atoms: from α- and β-phosphate of GDP, the backbone carbonyl group of Gly39, the side-chain carbonyl group of Asp12, and two sulfate ions present in the active site in the places of Had and 6-P_i_ group of 6-P-IMP. GDP is bound to residues Asp12-Gly16, Thr41, Lys322, Asp324, Ser400-Ser402, and calcium ion, as in the 6ZXQ structure. The hydrogen bond to the 6-P_i_ group of 6-P-IMP is replaced with the H-bond to sulfate ion present in its place, and interactions with Had and residues in the Asp-binding loop are, of course, missing. Accordingly, IMP retained most of the connections that involve its base and sugar parts (to residues: Arg135 from the adjacent monomer, Asn37, Gly119, Thr121, Thr230, and Val264), while the H-bond with Arg294 is missing. The sulfate ion present in the place of the 6′-P_i_ group of 6-P-IMP is extremely well anchored in the active site, maintaining all of the interactions that the 6-P_i_ group of 6-P-IMP realized [18], plus forming additional bonds with the O6 and N1 atoms of IMP. The sulfate ion present in the Asp-binding site interacts with the O6 atom of IMP, calcium ion, main chain and side chain of Thr290, and several water molecules.

The buried surface area between monomers in the structure of N-His-AdSS is 2422 Å^2^ (as calculated with Pisa, version 1.52 [23]), which compares well with 2342 Å^2^ calculated for the structure of C-His-AdSS [18]. The number of H-bonds and salt bridges at the interface is also almost the same: 27 + 4 and 26 + 4 for the N-His-AdSS structure and 6ZXQ, respectively.

## 3. Discussion

We have previously purified and characterized adenylosuccinate synthetase from *Helicobacter pylori* with His_6_-tag on its C-terminus (C-His-AdSS), and it showed properties similar to the wild-type enzyme [18]. We created the same enzyme with a His_6_-tag on its N-terminus (N-His-AdSS) as well. Even though this enzyme variant has the thrombin recognition site, we decided to keep the tag and check whether the position of the tag influences enzyme behavior.

The same purification procedure was used for C-His-AdSS and N-His-AdSS, namely affinity chromatography (AC) and size exclusion chromatography (SEC). However, as 20 additional amino acids in N-His-AdSS raise its pI compared to C-His-AdSS (as calculated by ProtParam tool, Table 1), the pH of the buffer for AC was lowered for this enzyme variant not to coincide with the protein’s pI, within the pH range suggested by the affinity resin producer. The molecular weight of monomer units estimated from the SDS-PAGE gels corresponds very well with the calculated one for both protein variants. However, elution volumes from SEC correlate to molecular weight (MW) of 66–68 kDa, i.e., between a monomer and a dimer. The shape of SEC peaks is not perfectly symmetrical, probably due to the high protein concentrations in the samples loaded (>60 mg/mL). A very similar elution volume (~16.30 mL, corresponding to ~76 kDa) was observed for C-His-AdSS loaded in lower concentrations. It is known that AdSS exists in solution in a dynamic equilibrium of a monomer and a dimer and that the biologically active form is a dimer. Moreover, the enzyme may be activated by substrate-induced dimerization as the presence of ligands pushes the equilibrium to the dimeric form [19,24]. Analytical ultracentrifugation measurements of WT and C-His-AdSS proved the existence of both monomers and dimers in thusly purified samples, which explains these SEC results [17,18]. Detailed AUC measurements of N-His-AdSS were conducted in this study, confirming also the case of this variant equilibrium between dimers and monomers, thus explaining observed elution volume (but see also below).

The predicted and experimentally confirmed pIs of three studied *H. pylori* AdSS variants, namely 7.94, 7.22, and 7.53 for N-His, C-His, and WT AdSS, respectively, are significantly higher than those of other bacterial AdSS enzymes, which are mostly between 5 and 6 [19]. Interestingly, the predicted pI of wild-type *H. pylori* AdSS (Table 1) is very similar to the pI of *Plasmodium falciparum* AdSS (pI 7.54). These parasitic protozoa also obtain their purine nucleoside supply through the purine salvage pathway and lack the de novo purine synthesis pathway [25].

Already during the purification, protein precipitation was observed in the N-His-AdSS samples, indicating some kind of protein instability. Indeed, temperature stability measurements showed drastically lowered stability of N-His-AdSS, compared to C-His and WT variants, namely 35% loss of activity at 30 °C and almost 100% loss at 37 °C. It has already been observed that some proteins have lower stability with His-tag attached [9]. Additionally, the different effect of His-tag on protein stability depending on its fusion site was observed for several proteins, like UbcA1 enzyme from the mushroom *Agrocybe aegerita* [26] or phenylacetone monooxygenase from *Thermobifida fusca* [27]. Dynamic light scattering (DLS) was employed to check the homogeneity and aggregation state of the purified AdSS variants. The DLS is a well-established method for the detection of aggregates [28] and it is often used to check the homogeneity of protein preparation, e.g., for crystallization, and in quality assessment of biopharmaceuticals [29]. This method confirmed differences amongst studied AdSS variants. Bimodal size distributions were observed only in the samples of N-His-AdSS, while C-His-AdSS and WT AdSS samples contained only monomodal species. Interestingly, MW estimated from the hydrodynamic radius corresponds to AdSS monomers. Having in mind the results from SEC, one possible explanation, confirmed by AUC experiments, is that in the conditions of the DLS measurements, performed with 150 mM NaCl, for C-His-AdSS and WT AdSS the equilibrium between monomer and dimer is shifted towards the monomer. In any case, the combination of bimodal size distribution and high polydispersity index (0.9) observed in DLS clearly shows the propensity of N-His-AdSS, unlike the other two enzyme variants, to form larger aggregates (therefore making the sample more heterogeneous), probably leading to protein precipitation.

Also, AUC experiments, which unlike SEC, DLS, and DSF last hours rather than minutes, clearly document differences in the oligomeric properties of N-His-AdSS compared to C-His- and WT AdSS (Figure 4). The AUC results show that only N-His-AdSS tends to aggregate and precipitate. Apparently, at first glance, N-His-AdSS looks in the AUC experiment to exist almost exclusively in the form of a biologically active dimer. However, quantitative analysis shows that a large fraction of the protein is missing from the sample. It probably, in agreement with DLS results, aggregated and precipitated out of the solution. By contrast, C-His- and WT AdSS are detected by AUC as a mixture of monomers and dimers, in the case of C-His-AdSS in some conditions with only a small admixture of larger but dissolved aggregates (Figure 4, middle left panel). Taken together, the SEC, DLS, DSF, and AUC results suggest that the His-tag located on the N-terminus of *H. pylori* AdSS makes the monomer of this enzyme prone to aggregation, which consequently leads to the precipitation of a significant fraction of the enzyme from the solution already within a few hours.

Specific activity and kinetic constants were determined for N-His-AdSS in the same conditions as before for C-His-AdSS and WT AdSS. Despite the fact that specific activity (and, related to it, maximal reaction velocity) is significantly lower for N-His-AdSS than for C-His-AdSS, Michaelis–Menten constants for all three substrates are in the same range for all three *H. pylori* AdSS variants (Table 4), and similar to those measured for other bacterial AdSS enzymes [30]. Even though *K*_m_ and *K*_d_ for an enzyme–substrate pair are constants of different meanings (kinetic vs. thermodynamic parameter, respectively [31]), similar values of *K*_m_ suggest that substrates bind to their respective active sites with similar affinity. Lower activity of the N-His-AdSS can be the result of several factors. For one, if the sample comprises a portion of enzyme molecules in aggregates, the population of catalytically active molecules is reduced. This is consistent with the SEC, DLS, DSF, and AUC results. However, the 20 residues long addition on the N-terminus, which is close to the dimerization domain, could not only make a monomer more prone to aggregation but could possibly also interfere with the productive binding of monomers thusly also reducing the population of the active dimeric enzyme molecules. Finally, this N-terminal addition is long enough that it could possibly impede the binding of substrates to the enzyme. As it is known that AdSS enzymes have the so-called P-loop involved in the binding of GTP close to the N-terminus [19], maybe the observed behavior of N-His-AdSS could be due to binding of His_6_-tag handle to or close to the GTP-binding site, hence disturbing the binding of this substrate to the active site. For example, Given and coworkers recently discovered the N-terminal His-tag handle (comprising also protease cleavage site) bound in the active site in the 3D structure of *Geobacillus stearothermophilus* purine nucleoside phosphorylase [32]. While the enzyme in solution retained some of its activity, the His-tag handle was mostly uncleavable, indicating competition between the substrate and the handle in solution, with most of the enzyme population in the handle-bound state [32]. However, similar Michaelis–Menten constants observed for all three *H. pylori* AdSS variants suggest that such competition does not occur in the case of N-His-AdSS.

Nevertheless, to examine whether there is any structural change that could explain the propensity of N-His-AdSS to aggregate and/or its lower activity, the structural features of this enzyme variant were investigated by circular dichroism (CD) and X-ray diffraction studies. The CD was employed to check the secondary structure of all investigated *H. pylori* AdSS variants in solution. Already by inspection of the CD spectra, it could be noticed that all three variants of the enzyme share a similar secondary structure (Figure 5). The calculation of shares of individual secondary structure elements confirmed this observation (Table 5). Thus, it seems that the N-His handle does not significantly affect the secondary structure composition of N-His-AdSS, at least in the conditions used.

The determined 3D structure of N-His-AdSS is similar to all AdSS structures solved so far [19], forming a biologically functional dimer with the side chain of Arg135 participating in building the active site of the neighboring monomer. Comparison of the N-His-AdSS structure obtained now with the fully ligated structure of C-His-AdSS (6ZXQ) showed that the only difference is in the position of the Asp-binding loop (residues 289–294), and the overall RMSD for these two structures is only 0.229 Å (for 377 atoms). As there is no hadacidin observed in the Asp-binding site in the structure of N-His-AdSS, this loop is not anchored and has more freedom to move. A similar situation was observed in the structure of C-His-AdSS in a complex with IMP only (7PVO [18]). Both structures, of N-His-AdSS, determined in this study, and of C-His-AdSS with IMP only (7PVO), have IMP tilted with respect to the 6-P-IMP present in the 6ZXQ structure, Had/Asp in the active site replaced by a sulfate ion, and Asp-binding loop moved away from the active site. RMSD is 0.234 Å (for 342 atoms) between N-His-AdSS and 7PVO structures. From the 6ZXQ structure, it was concluded that during incubation/crystallization, the first step of the enzymatic reaction occurred. Namely, the phosphate group was transferred from GTP to IMP, forming 6-P-IMP, as suggested previously by Soans [33], but the reaction could not continue without the third substrate, Asp [18]. However, in the structure presented here, GTP is also found to be hydrolyzed, but no 6-P-IMP is formed. The same situation is observed in the 3D structure of AdSS from the fungus *Cryptococcus neoformans* (PDB code 5I34 [34]). Authors suggested several possible explanations: the AdSS-IMP-GTP/GDP complex was captured in a state of flux between the two binding states, GTP was hydrolyzed to GDP before crystal formation began and GDP bound to the GTP binding pocket anyway, or GTP was hydrolyzed in the time it took for crystals to form [34]. Considering that it is not possible to determine which of these events occurred, that several moieties of the same shape, consistent with phosphate and sulfate ions, were observed in the presently solved N-His-AdSS structure even outside of the active site, that there is ammonium sulfate present in the crystallization conditions, and that there is no crystallographic way of distinguishing phosphate group from sulfate group, it is most likely that all these detected moieties are sulfate ions. Since no His_6_-tag handle is visible in the N-His-AdSS structure, it can be concluded that it is too flexible to be observed, and obviously not so firmly bound to the protein as in the mentioned case of *G. stearothermophilus* purine nucleoside phosphorylase [32]. This, however, does not preclude the possibility that in solution the handle affects the behavior of the monomer of this enzyme variant, making it prone to aggregation, and disturbs functional dimerization of N-His-AdSS, as the N-terminus of this protein is near the dimerization interface (Figure 6).

In conclusion, our results demonstrate that the His_6_-tag located at the N-terminus of the *H. pylori* AdSS enzyme, biologically active as a homodimer, makes the monomer of this enzyme prone to aggregation. This, in turn, significantly influences the equilibrium of the oligomeric forms of N-His-AdSS present in the solution and slightly lowers its activity, unlike the tag located at the other end of this enzyme. Thus, *H. pylori* AdSS makes another example that reinforces the need to check the influence of the attached tag, as well as the site of its attachment, on the properties of the investigated protein, especially an oligomeric one.

## 4. Materials and Methods

### 4.1. Purification of Enzymes

We have previously created plasmid *pET21b-HPpurA-CHis*, bearing gene coding for *H. pylori* AdSS including His_6_-tag on the C-terminus [18]. Plasmid *pET28c-HPpurA-NHis*, bearing gene coding for *H. pylori* AdSS including His_6_-tag on N-terminus was created by the PCR reaction: a fragment of the *hp0255* gene (*purA*—the gene encoding the AdSS protein, GenBank ID: AAD07324.1) was amplified using a pair of primers with additional flanking regions for NdeI and XhoI restriction sites on the template of genomic DNA isolated from the reference *H. pylori* strain 26695. Primer NdeI-purA: 5′-GCGTTGATCATATGGCAGATGTCGTTGTGGG-3′, primer XhoI-purA: 5′-GGAGGATCTCGAGTCATAGAAAAATCGTGTCTTCTCTTTCAGG-3′. The PCR reaction was performed using PrimeSTAR DNA polymerase (Takara Bio, Kusatsu, Japan) under the following conditions: 4 min at 98 °C, followed by 30 cycles of denaturation at 98 °C for 10 s, annealing at 59 °C for 15 s, and elongation at 72 °C for 1.5 min. The final elongation step was performed at 72 °C for 10 min. The *purA* gene fragment (1236 kb) obtained as a result of the PCR reaction was isolated from a 0.8% agarose gel, and then the ligation reaction was performed with the *pET28c* plasmid vector (5367 bp) (previously digested with the same NdeI and XhoI restriction endonucleases) in order to obtain *pET28c-HPpurA-NHis* plasmid. Both plasmids were (separately) subcloned by electroporation into *E. coli* cell strain BL21-CodonPlus(DE3)-RIL (Agilent Biotechnology, Santa Clara, CA, USA), which was used for the expression of enzymes. Expression was performed as before for C-His-AdSS [18], for C-His-AdSS in the presence of 100 µg/mL ampicillin, and for N-His-AdSS in the presence of 50 µg/mL kanamycin.

Enzyme purification proceeded at +4 °C in the same manner (affinity chromatography and size exclusion chromatography) as before for C-His-AdSS [18], with minor modifications. Namely, affinity chromatography buffers used for N-His-AdSS were of pH 7.5, while those for C-His-AdSS were of pH 8.0. Also, the elution from the Ni-NTA affinity column was performed with an imidazole gradient of 10–500 mM in 22 mL. Superdex 200 Increase 10/300 GL column (Cytiva Life Sciences, Marlborough, MA, USA) was used for the size exclusion chromatography (SEC), run in 20 mM Hepes-NaOH pH 7.0 buffer containing 150 mM NaCl and 1 mM 2-mercaptoethanol (SEC buffer), at 0.5 mL/min, on the ÄKTA pure protein purification system (Cytiva Life Sciences). This column was calibrated with several proteins from the Gel Filtration Calibration Kit (Cytiva Life Sciences)—chymotrypsinogen A (25 kDa), yeast alcohol dehydrogenase (37.5 kDa), bovine serum albumin (67 kDa), catalase (232 kDa) and ferritin (440 kDa), and void volume was determined with Blue Dextran. The elution volume of the marker proteins was determined by absorption detection at 280 nm. The wild-type variant of AdSS was purified as described before, by the ion exchange chromatography and the size exclusion chromatography [17].

The purification was monitored by electrophoresis under denaturing conditions (SDS-PAGE) and a protein concentration assay following the method of Bradford [35] with bovine serum albumin as a standard. For purified protein samples, protein concentration was determined utilizing protein’s molar extinction coefficient at 280 nm of 38,850 M^−1^ cm^−1^ (as calculated by the ProtParam tool at ExPasy.org, the same for all investigated protein variants).

The purified SEC fractions of AdSS variants (10–15 mg/mL in SEC buffer) were stored in aliquots at −80 °C until use.

### 4.2. Electrophoretic Techniques

Electrophoreses were run on a PhastSystem apparatus (Cytiva Life Sciences), with precast gels, according to manufacturer’s instructions. For electrophoresis under denaturing conditions (SDS-PAGE), PhastGel Homogeneous 12.5 plates were used, with PhastGel Buffer strips, SDS. For isoelectric focusing (IEF), PhastGel IEF 3–9 plates were used. Prior to loading on the SDS-PAGE gels, samples were mixed with the treatment buffer (20 mM Tris-HCl pH 8.0, 2 mM ethylenediaminetetraacetic acid (EDTA), 5% sodium dodecyl sulfate (SDS), 10% 2-mercaptoethanol) in the ratio 1:1 (*v*/*v*) and heated for 10 min at 98 °C. Samples were loaded on the IEF gels without any treatment. Gels were stained with Coomassie Brilliant Blue R-250. Marker protein mixtures were loaded with samples—LMW-SDS Marker Kit (14.4–97 kDa, Cytiva Life Sciences) or IEF Marker 3–10 (SERVA Electrophoresis GmbH, Heidelberg, Germany).

### 4.3. Enzyme Activity Assay

Enzyme activity assay was performed spectrophotometrically, as described before [17], in 1 mL reaction mixture in 20 mM Hepes-NaOH buffer pH 7.7, at room temperature (25 °C), with saturating substrate concentrations (guanosine triphosphate, GTP at 0.06 mM, inosine monophosphate, IMP at 0.15 mM and sodium aspartate, Asp at 5 mM) and 1 mM MgCl_2_, utilizing molar extinction coefficient at 280 nm of 1.17 × 10^4^ M^−1^ cm^−1^ (formation of adenylosuccinate [36]).

One unit (U) of AdSS enzymatic activity is defined as the amount of the enzyme necessary to catalyze the formation of one µmol of adenylosuccinate per min at 25 °C, in given conditions. Specific activity is expressed as units per mg of protein (U/mg).

When N-His-AdSS was tested for pH and temperature stability, the enzyme was incubated in 0.5 mg/mL concentration, either at different temperatures in 20 mM Hepes-NaOH buffer pH 7.7 or in Britton–Robinson universal buffer of different pH [37] at 25 °C. After one hour, 4 µL of the incubation mixture was used for each activity measurement under standard conditions (20 mM Hepes-NaOH buffer pH 7.7, at 25 °C, with saturating substrate concentrations).

### 4.4. Determination of Enzyme Kinetic Constants

Kinetic constants of *H. pylori* N-His-AdSS were determined spectrophotometrically, in the same manner as for the WT AdSS and C-His-AdSS [17,18]. Briefly, enzyme activity was measured while the concentration of one substrate was varied, and concentrations of the other two substrates were kept constant (GTP at 0.06 mM, IMP at 0.15 mM, and Asp at 5 mM), in 20 mM Hepes-NaOH buffer pH 7.7, at room temperature (25 °C). The experimentally obtained data were analyzed by fits of the Michaelis–Menten equation using GraphPad Prism version 10.0 (GraphPad Software, Inc., San Diego, CA, USA).

### 4.5. Dynamic Light Scattering (DLS) Measurements

DLS measurements were performed at 4 °C in the SEC buffer using a Zetasizer Nano ZS instrument (Malvern Instruments, Malvern, UK) with a green laser (532 nm), set at an angle of 173°. AdSS concentration was 1 mg/mL. The hydrodynamic diameter (dh) of particles was determined as the value of the peak maximum of the volume and intensity size distributions and is given as an average of 6 measurements. All samples were measured in disposable ZEN 0040 cuvettes. Data processing was carried out with Zetasizer software version 8.02 (Malvern Instruments, Malvern, UK).

### 4.6. Low-Volume Differential Scanning Fluorimetry (nanoDSF)

Nano DSF measurements were performed using the Prometheus NT.48 nanoDSF device (NanoTemper Technologies, Munich, Germany) as previously described [18]. The assay was carried out in 20 mM Hepes-NaOH buffer pH 7.7, in the presence of different NaCl concentrations. The samples were loaded into Prometheus Standard Capillaries. Thermal unfolding was monitored in 20–80 °C range, using a linear thermal ramp (1 °C·min^–1^). The experimental data were analyzed with the implemented software. The middle-point temperature of thermal unfolding (T_m_) was estimated based on the location of the maximum of the first derivative of the fluorescence intensity 350 nm/330 nm ratio. Additionally, using the back reflection optics, based on an induced scattering of light, aggregation of protein samples was detected. For each condition tested, the average of at least six experiments is presented.

### 4.7. Analytical Ultracentrifugation Experiments (AUC)

AUC sedimentation velocity measurements with absorbance detection were completed as previously described [17,18]. Optima XL-I ultracentrifuge (Beckman-Coulter Inc., Indianapolis, IN, USA), equipped with An-50Ti and An-60Ti analytical rotors, and double-sector 1.2-cm cells with Epon-charcoal centerpieces with sapphire or quartz windows was used. The partial specific volumes of AdSS variants, based on their amino acid composition, as well as densities and viscosities of buffers, were calculated using the Sednterp program (version 1.09) [38]. Radial scans of the absorption profiles in the cell were measured at 5- or 7-min intervals on the same protein samples. Analysis of the ultracentrifugation data was completed with the Sedfit program (version 16.1c) using the continuous c(s) distribution model [39]. Both the sedimentation coefficient, s, and the standard sedimentation coefficient, s^0^_20,w_, were calculated, and based on them the molecular mass of the species present in the solution was determined, assuming that they have the same friction ratio (also obtained as a parameter in the Sedfit program). To compare the influence of a particular parameter (enzyme concentration, presence of salt) all three enzyme variants were spun simultaneously to secure exactly the same conditions.

### 4.8. Enzyme Secondary Structure by Circular Dichroism (CD)

CD spectra of sample proteins (in concentration of 1 mg/mL) were taken on UV-CD-spectrometer Jasco J-815 (JASCO, Easton, MD, USA) with the following parameters: cuvette 0.02 cm, selectivity standard, data pitch 0.2 nm, bandwidth 1 nm, response 1 sec, measurement range 260–190 nm, accumulation 2, scanning speed 50 nm/min, temperature 25 °C. The spectra were corrected by subtractions of the baseline made with SEC buffer. The spectra are represented as average MRE (Molar residual ellipticity/deg cm^2^ dmol^−1^) values. Average spectra were calculated from two (WT AdSS) or three (C-His-AdSS, N-His-AdSS) replicas. The spectra were drawn and smoothed in the OriginPro7.5G software (OriginLab, Northampton, MA, USA) using the adjacent averaging method in 25 points. Secondary structure of proteins was calculated from the averaged data using the BeStSel online software (https://bestsel.elte.hu, accessed on 28 April 2024) [22]. The HT voltage applied to detector for all the data used for MRE measurement is lower than 600 V.

### 4.9. Enzyme Crystallization, Data Collection, and 3D Structure Determination

Purified N-His-AdSS (9.25 mg/mL) was incubated with MgCl_2_, GTP, IMP, and hadacidin (all ligands in 10-fold molar excess) for 15 min at room temperature. Crystallization trials were set up at 18 °C by the hanging drop vapor diffusion method (volumes: 2 µL drop with the 1:1 ratio, 700 µL well). Conditions that previously gave the best crystals of C-His-AdSS, namely 20% PEG3350, 0.2 M ammonium sulfate, 0.1 M Tris-HCl pH 8.5, were tested, along with the same precipitants in 0.1 M Tris-HCl pH 6.8. Crystals appeared after a few days in conditions with lower pH, they were pulled through solution of 20% glycerol in mother liquor and cooled in liquid nitrogen. X-ray diffraction data collection from several crystals was completed at the synchrotron beamline Elettra (XRD2, Trieste, Italy). The data collection and refinement parameters for the best data set are summarized in Table 6. The data were processed using the XDS program (version June 30, 2023) [40]. The structure was solved by molecular replacement using the MOLREP program (version 11.0) [41] and the structure of fully ligated C-His-AdSS (PDB code 6ZXQ) as a search template. The model was refined using the phenix.refine routine from the PHENIX package (version 1.19) [42]. Coordinates and structure factors have been deposited in the Protein Data Bank under the accession number 9F17.

## Figures and Tables

**Table 1:** Basic properties of adenylosuccinate synthetase (AdSS) from *H. pylori*, variants with His_6_-tag on N- and C-terminus compared to the wild-type, calculated with ProtParam tool on ExPasy.org.

Enzyme Variant	N-His-AdSS	C-His-AdSS	WT AdSS
Addition to the sequence	MGSSHHHHHHSSGLVPRGSH-	-LEHHHHHH	-
Number of residues	431	419	411
Molecular weight (MW)	47,906.04	46,807.84	45,742.72
Isoelectric point (pI)	7.94	7.22	7.53
Extinction coefficient (M^−1^ cm^−1^, at 280 nm)	38,850	38,850	38,850
Instability index	28.66	28.02	28.55

**Table 2:** Thermal stability characterized by nanoDSF measurements of adenylosuccinate synthetase (AdSS) from *H. pylori*, variants with His_6_-tag on N- and C-terminus compared to the wild-type enzyme: the middle-point temperature of the thermal unfolding (T_m_) and the onset aggregation temperature (T_turbidity_).

NaCl (mM)	Protein Concentration Range (µM)	WT AdSS		C-His-AdSS		N-His-AdSS	
		T_m_ (°C)	T_turbidity_ (°C)	T_m_ (°C)	T_turbidity_ (°C)	T_m_ (°C)	T_turbidity_ (°C)
0	1–3	39.2 ± 1.6	36.2 ± 2.8	32.0 ± 0.7	30.9 ± 0.7	33.1 ± 1.1	29.9 ± 1.4
0	8–10	40.9 ± 1.7	35.8 ± 1.6	36.0 ± 0.3	31.1 ± 0.7	38.9 ± 1.6	27.5 ± 1.6
150	1–20	48.7 ± 0.2	42.0 ± 1.6	46.9 ± 0.3	42.4 ± 1.3	47.1 ± 0.5	39.9 ± 2.3
300	1–20	50.7 ± 0.2	43.3 ± 3.0	49.0 ± 0.2	44.8 ± 2.0	49.3 ± 0.4	43.2 ± 1.6

**Table 3:** Distribution of *H. pylori* AdSS, as a function of concentration, between monomeric and oligomeric forms, c(s), sedimentation coefficients, s_20,w_, and estimated molecular masses, MW, of these forms, obtained from the analytical ultracentrifugation studies, with and without 150 mM NaCl.

NaCl (mM)	Protein (µM)	WT AdSS			C-His-AdSS			N-His-AdSS		
		s_20,w_^a^ (S)	MW ^b^ (kDa)	c(s)	s_20,w_^a^ (S)	MW ^b^ (kDa)	c(s)	s_20,w_ ^a^ (S)	MW ^b^ (kDa)	c(s)
0	1.30–1.53	3.79	41.4	0.07	3.78	42.5	0.15	----	----	----
		5.53	73.0	0.33	5.80	80.8	0.24	5.70	94.6	0.13
150	1.30–1.53	4.08	42.3	0.30	3.92	31.2	0.09	----	----	----
		----	----	----	----	----	----	5.54	88.4	0.15
0	2.73–5.98	3.88	42.2	0.06	3.94	36.9	0.30	3.82	40.2	0.05
		5.70	75.2	0.67	5.55	61.6	0.60	5.73	73.7	0.31
150	2.73–5.98	3.94	43.2	0.19	4.10	70.6	0.63	3.54	36.6	0.01
		5.32	67.7	0.74	----	----	----	5.97	79.9	0.51

^a^ Theoretical value of the sedimentation coefficient, s_20,w_ of *H. pylori* AdSS monomer is s_monomer_ = 3.53 S, the value calculated with the Hydropro program (version 10) [20] using the PDB 7PVO structure of this enzyme [18], while sedimentation coefficient for a dimer is s_dimer_ = 5.60 S as calculated according to the formula: s_dimer_ = s_monomer_ 2 ^2/3^ [21], assuming that the value of frictional ratio of both oligomeric forms is the same. ^b^ MW values in the table, calculated from sedimentation coefficients, are in most cases only a rough estimation of molecular masses because peaks of c(s) distribution are often not symmetrical, and have low intensity or overlap (see Figure 4).

**Table 4:** The kinetic parameters for N-His-AdSS from *H. pylori*, obtained by fitting the Michaelis–Menten equation to the experimental data. Fitting errors of the kinetic parameters obtained are shown in the table. Data for WT AdSS are from reference [17] and for C-His-AdSS from reference [18]. Fixed substrate concentrations: 5 mM Asp, 0.15 mM IMP, 0.06 mM GTP.

	Variable Substrate	*K_m_* (µM)	*V_max_* (U/mg)
N-His-AdSS	Asp	90.1 ± 13.0	0.331 ± 0.014
	IMP	21.7 ± 2.4	0.421 ± 0.014
	GTP	11.9 ± 1.3	0.391 ± 0.011
C-His-AdSS	Asp	176.3 ± 18.9	0.894 ± 0.030
	IMP	35.9 ± 4.6	0.956 ± 0.040
	GTP	15.6 ± 1.9	1.103 ± 0.040
WT AdSS	Asp	125.4 ± 7.7	1.103 ± 0.016
	IMP	40.1 ± 2.9	1.456 ± 0.036
	GTP	8.7 ± 0.6	1.418 ± 0.023

**Table 5:** Calculated secondary structure elements content (obtained with the BeStSel software) for averaged CD spectrum of *H. pylori* N-His-AdSS and C-His-AdSS in comparison to WT AdSS.

Secondary Structure Element/%	Protein Variant		
	WT AdSS	C-His-AdSS	N-His-AdSS
α-helix	25.4	27.0	26.4
β-strand—antiparallel	16.6	11.7	14.5
β-strand—parallel	9.3	7.1	8.0
Turn	9.9	10.4	11.7
Other	38.9	43.7	39.4

**Table 6:** Data collection and refinement statistics.

	N-His-AdSS
Wavelength (Å)	1.0
Resolution range (Å)	44.12–1.7 (1.761–1.7) ^†^
Space group	*P*2_1_
Unit cell (Å)	69.15 122.7 70.15 90 113.355 90
Total reflections	763,453 (73,588)
Unique reflections	117,404 (11,577)
Multiplicity	6.5 (6.4)
Completeness (%)	99.70 (98.59)
Mean I/sigma (I)	13.26 (1.14)
Wilson B-factor	24.46
*R_merge_*	0.09438 (1.654)
*R_meas_*	0.1027 (1.803)
*R_pim_*	0.04001 (0.7073)
CC_1/2_	0.999 (0.585)
CC*	1 (0.859)
Reflections used in refinement	117,305 (11,551)
Reflections used for *R_free_*	5721 (550)
*R_work_*	0.1777 (0.3225)
*R_free_*	0.2093 (0.3590)
CC (work)	0.971 (0.774)
CC (free)	0.959 (0.710)
Number of non-hydrogen atoms	7460
Macromolecules	6498
Ligands	127
Solvent	835
Protein residues	826
RMS (bonds)	0.010
RMS (angles)	1.03
Ramachandran favored (%)	97.08
Ramachandran allowed (%)	2.55
Ramachandran outliers (%)	0.36
Rotamer outliers (%)	0.71
Clashscore	4.83
Average B-factor	32.73
Macromolecules	31.69
Ligands	35.87
Solvent	40.74

^†^ Statistics for the highest-resolution shell are shown in parentheses.

## Data Availability

Data is contained within the article and Supplementary Materials.

## Supplementary Materials

The following supporting information can be downloaded at: https://www.mdpi.com/article/10.3390/ijms25147613/s1.
