# Supplementary material for: Location Is Everything: Influence of His-Tag Fusion Site on Properties of Adenylosuccinate Synthetase from Helicobacter pylori
Source: Int J Mol Sci. 2024 Jul 11;25(14):7613. doi: 10.3390/ijms25147613 (PMC11276676; doi:10.3390/ijms25147613)
Supplement: Supplementary file 1 [file ijms-25-07613-s001.zip › ijms-3014160-supplementary.pdf]

Supplemental information

Location is everything: Influence of His-tag fusion site on properties of adenylosuccinate synthetase from *Helicobacter pylori*

Marija Zora Mišković [1,†], Marta Wojtyś [2,†], Maria Winiewska-Szajewska [2,3], Beata Wielgus-Kutrowska[2], Marija Matković [4], Darija Domazet Jurašin [5], Zoran Štefanić [5], Agnieszka Bzowska [2,*] and Ivana Leščić Ašler [5,*]

[1] Department of Chemistry, Faculty of Science, University of Zagreb, Horvatovac 102a, HR-10000 Zagreb, Croatia; mmiskovic@stud.biol.pmf.hr
[2] Division of Biophysics, Institute of Experimental Physics, Faculty of Physics, University of Warsaw, Pasteura 5, 02-093 Warsaw, Poland; mi.wojtys@fuw.edu.pl, maria.winiewska@fuw.edu.pl, beata.wielgus-kutrowska@fuw.edu.pl, agnieszka.bzowska@fuw.edu.pl
[3] Institute of Biochemistry and Biophysics, Polish Academy of Sciences, Pawinskiego 5a, 02-106 Warsaw, Poland; maria.winiewska@fuw.edu.pl
[4] Division of Organic Chemistry and Biochemistry, Ruđer Bošković Institute, Bijenička cesta 54, HR-10000 Zagreb, Croatia; marija.matkovic@irb.hr
[5] Division of Physical Chemistry, Ruđer Bošković Institute, Bijenička cesta 54, HR-10000 Zagreb, Croatia; darija.domazet.jurasin@irb.hr, zoran.stefanic@irb.hr, ivana.lescic.asler@irb.hr
[†] These authors contributed equally to this work
* Correspondence: ILA - ivana.lescic.asler@irb.hr; Tel.: +385-1-4561111
AB - agnieszka.bzowska@fuw.edu.pl; Tel.: +48-22-5532341

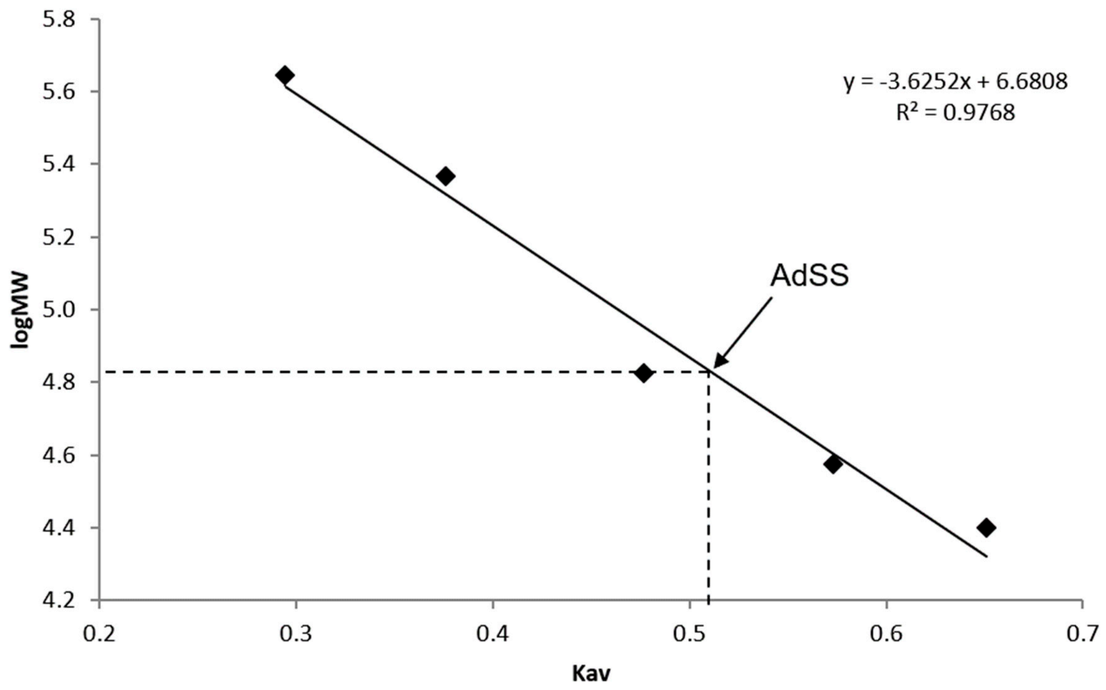

Figure S1. Estimation of *H. pylori* AdSS molecular weight by size-exclusion chromatography on Superdex 200 Increase 10/300 GL column (Cytiva Life Sciences). Calibration curve for the used column, using several proteins from the Gel Filtration Calibration Kit (Cytiva Life Sciences) – chymotrypsinogen A (25 kDa), yeast alcohol dehydrogenase (37.5 kDa), bovine serum albumin (67 kDa), catalase (232 kDa) and ferritin (440 kDa), and void volume determined with Blue Dextran. Average distribution constant, K_{av} was calculated as $K_{av}=(V_e-V_0)/(V_c-V_0)$, where V_e is the elution volume, V_0 – void volume of the column (9.13 mL), V_c – geometrical bed volume of the column (23.6 mL). V_e(N-His-AdSS) = 16.54 mL, V_e(C-His-AdSS) = 16.50 mL.

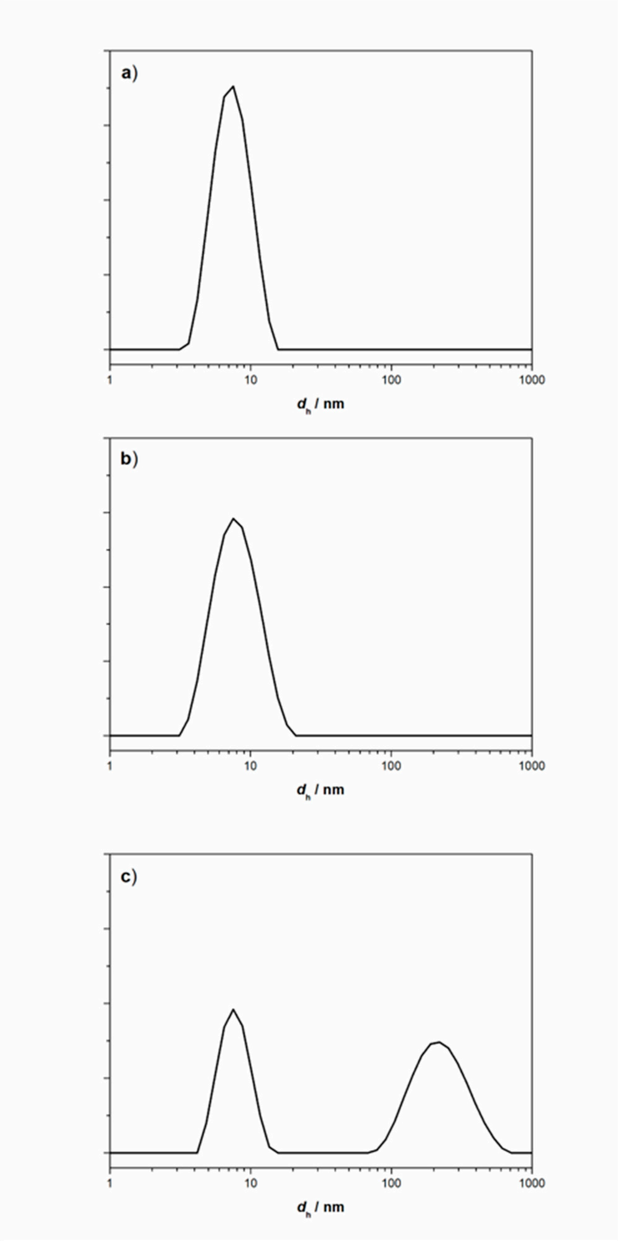

Figure S2. Results of the representative DLS experiment showing the intensity size distributions for 1 mg/mL suspensions of three variants of *H. pylori* AdSS in 20 mM Hepes-NaOH pH 7.0 buffer containing 150 mM NaCl and 1 mM 2-mercaptoethanol (SEC buffer), taken at 4 °C: **(a)** WT, **(b)** C-His and **(c)** N-His.
